# Letter to the Editor Regarding “Treatment Pathways and Outcomes in Patients with BMI ≥ 50 kg/m^2^: Conservative Treatment, Immediate Surgery or Stepwise Surgical Approach”

**DOI:** 10.1007/s11695-025-08157-0

**Published:** 2025-08-12

**Authors:** Shuting Yin, Jiping Chen, Wei Wei

**Affiliations:** 1 Department of General Surgery, the Second Affiliated Hospital of Nanjing Medical University, Nanjing, China; 2 Department of Esophageal Surgery and Thoracic Surgery, Nanjing Drum Tower Hospital, The Affiliated Hospital of Nanjing University Medical School, Nanjing, China

Dear Editor,

We read with great interest the article by Sara Notz et al., titled “Treatment Pathways and Outcomes in Patients with BMI ≥ 50 kg/m^2^”, published in Obesity Surgery. We commend the authors for focusing on patients with BMI ≥ 50 kg/m^2^, a particularly high-risk and under-researched population, and for providing real-world data to inform treatment decisions [1].

This single-center retrospective study included 538 patients, who were retrospectively assigned to three groups according to treatment strategy for comparative analysis. The results suggest that immediate metabolic surgery (“Surg-First”) is associated with greater weight loss, better resolution of comorbidities, and lower complication rates than a stepwise approach. These findings support the feasibility of a “surgery-first” strategy for patients with BMI ≥ 50 kg/m^2^.

While we recognize the study’s strengths, we offer several clinical and statistical considerations and recommend cautious interpretation of the claim that immediate surgery is superior to a stepwise approach.

Non-Randomized Design and Potential Bias: Group allocation was primarily based on patient preference, insurance, and MDT decision-making rather than standardized criteria. This introduces potential selection bias. For example, the proportion of ASA IV patients was significantly higher in the Surg-First group (9.3%) than in the Step-Treat group (0.9%), yet major complications (Clavien-Dindo ≥ IIIb) were lower (3.6% vs. 11.8%). The authors suggest factors such as delayed treatment and psychological stress may explain this, but unmeasured confounders likely contributed. Use of methodological approaches such as inverse probability of treatment weighting (IPTW) could improve baseline comparability between groups and strengthen the scientific validity and causal interpretability of treatment pathway comparisons.

Residual Confounding Due to Missing Variables: Although Firth logistic regression was applied, several key confounders were not included in the multivariable model, such as obesity etiology (genetic, drug-induced, endocrine-related), duration of obesity, psychiatric history, socioeconomic status, adherence to conservative therapy, and surgical complexity indicators (e.g., liver volume, visceral fat). Without adjusting for these, the association between treatment strategy and surgical outcomes remains uncertain. A more comprehensive regression model, incorporating Cox proportional hazards for time-dependent outcomes, would better reflect the independent relationship between treatment pathways and clinical outcomes.

Overly Strict Definition of Conservative Success: The study defined successful conservative treatment as > 20% weight loss, based on the German S3 guideline, but did not account for stratified targets by BMI level, nor justify applying this high threshold to all patients with BMI ≥ 50 kg/m^2^. In contrast, major international guidelines (AHA/ACC, AACE, WHO) consider 5–10% weight loss sufficient for meaningful metabolic benefits [2]. The 20% cutoff exceeds widely accepted clinical standards. Additionally, The SF-BARI QoL score combines weight loss, complications, and subjective QoL, potentially causing overlap and interpretation bias. Separate reporting of individual outcomes is recommended.

Insufficient Long-Term Outcome Assessment: Although the study included 24 months of follow-up, long-term issues beyond two years—such as nutritional deficiencies, metabolic bone disease, sarcopenia, weight regain, and recurrence of comorbidities—are particularly relevant in patients with BMI ≥ 50 kg/m^2^. Postoperative lifestyle, medication adherence, and psychosocial factors may also influence outcomes but were not assessed. Future studies should incorporate body composition (e.g., DEXA), micronutrient levels, functional status, and psychological well-being to provide a more comprehensive view of recovery. This would support both outcome evaluation and postoperative education.

Limited Applicability to Broader Settings: This study was conducted at a single center in Germany, where sleeve gastrectomy (SG) was the preferred procedure. Surgical choices and cultural context may differ in regions favoring Roux-en-Y gastric bypass (RYGB). Moreover, healthcare systems and patient selection processes affect treatment access. While the findings are valuable, they may not be directly generalizable to other countries or healthcare settings. Multicenter studies with diverse populations are needed to improve external validity.

In conclusion, the study by Sara Notz et al. provides valuable clinical evidence on treatment strategies for patients with super obesity, particularly in supporting immediate metabolic surgery for those with BMI ≥ 50 kg/m^2^. However, due to limitations in group allocation, confounder adjustment, follow-up duration, and outcome definitions, the conclusion that immediate surgery is superior to a stepwise approach should be interpreted with caution. We look forward to future prospective, multicenter studies using propensity score–matched designs to validate these findings and optimize individualized management for this high-risk population.

We hope our comments contribute to further research and discussion in this important field.

## Data Availability

No datasets were generated or analysed during the current study.
